# Transfer of MicroRNAs by Embryonic Stem Cell Microvesicles

**DOI:** 10.1371/journal.pone.0004722

**Published:** 2009-03-06

**Authors:** Alex Yuan, Erica L. Farber, Ana Lia Rapoport, Desiree Tejada, Roman Deniskin, Novrouz B. Akhmedov, Debora B. Farber

**Affiliations:** Jules Stein Eye Institute, UCLA School of Medicine, Los Angeles, California, United States of America; University of Florida, United States of America

## Abstract

Microvesicles are plasma membrane-derived vesicles released into the extracellular environment by a variety of cell types. Originally characterized from platelets, microvesicles are a normal constituent of human plasma, where they play an important role in maintaining hematostasis. Microvesicles have been shown to transfer proteins and RNA from cell to cell and they are also believed to play a role in intercellular communication. We characterized the RNA and protein content of embryonic stem cell microvesicles and show that they can be engineered to carry exogenously expressed mRNA and protein such as green fluorescent protein (GFP). We demonstrate that these engineered microvesicles dock and fuse with other embryonic stem cells, transferring their GFP. Additionally, we show that embryonic stem cells microvesicles contain abundant microRNA and that they can transfer a subset of microRNAs to mouse embryonic fibroblasts *in vitro*. Since microRNAs are short (21–24 nt), naturally occurring RNAs that regulate protein translation, our findings open up the intriguing possibility that stem cells can alter the expression of genes in neighboring cells by transferring microRNAs contained in microvesicles. Embryonic stem cell microvesicles may be useful therapeutic tools for transferring mRNA, microRNAs, protein, and siRNA to cells and may be important mediators of signaling within stem cell niches.

## Introduction

Circulating platelet-derived vesicles were first identified in human plasma in the 1960's [1]. These vesicles, called microvesicles or microparticles, are heterogeneous in size and range from ∼30 nm to 1 μm. Previously believed to be inert cellular debris, microvesicles are now gaining acceptance as important mediators of intercellular communication [2]–[9]. For example, microvesicles may mediate intercellular communication by transporting bioactive lipids, mRNA, or proteins between cells. Microvesicles have been identified from many cellular sources including monocytes, macrophages, endothelial cells, leukocytes, polymorphonuclear leukocytes, and tumor cells [2], [4], [5], [10]. More recently, microvesicles were isolated from embryonic stem cells. These embryonic stem cell microvesicles (ESMVs) were capable of reprogramming hematopoietic progenitors [6], suggesting that microvesicles are capable of providing stem cells with extrinsic cues, which may regulate stem cell proliferation, and fate in stem cell niches. Shedding of microvesicles is a normal physiological process and is interestingly related to high rates of cellular proliferation. Cellular stress and damage, however, can also result in the release of membrane microvesicles [7], [11]–13. In humans, microvesicles play an essential role in maintaining hematostasis. A reduction in the number of platelet microvesicles causes a bleeding disorder called Scott Syndrome [14]. Elevated levels of microvesicles are also associated with a variety of disorders including acute coronary syndrome, hypertension, diabetes, and pulmonary embolism (reviewed in [15]). In addition to maintaining hematostasis, microvesicles have also been implicated in carrying membrane bound morphogens in *Drosophila* [9], and they may influence the behavior and survival of hematopoietic progenitors [6], [10].

The mechanism by which ESMVs may mediate intercellular signaling could involve the activation of receptors on the recipient cell by ligands in the ESMV. In this manner, ESMVs would be able to carry membrane bound ligands considerable distances from their stem cell origin. Alternatively, ESMVs may be able to mediate signaling by the direct transfer of proteins, RNA, or bioactive lipids to the recipient cell, serving as “physiological liposomes” [6], [7]. If ESMVs can indeed serve as “physiological liposomes,” transferring RNA and proteins to cells, they can perhaps be used to deliver exogenously expressed genes for therapeutic purposes. To explore this possibility, we first characterized the RNA and protein content of ESMVs and whether they carry the mRNA and protein expressed from a transgene present in embryonic stem cells (ESCs) encoding green fluorescent protein (GFP). We then investigated the ability of ESMVs to transfer GFP to recipient cells. We also explored the possibility that ESMVs can transport and transfer microRNAs (miRNAs) to cells. MiRNAs are a group of small (21–24nt) noncoding RNAs that regulate gene expression in mammals by binding to the 3′ untranslated region (UTR) of mRNAs to repress translation [16]–[18]. miRNAs have been found in peripheral blood microvesicles of healthy individuals [19] and in ovarian tumor microvesicles circulating in the peripheral blood of patients [20]. We show that ESMVs are capable of transferring a subset of miRNAs to mouse embryonic fibroblasts (MEFs), suggesting a tightly regulated transfer process. Transfer of miRNAs by microvesicles represents a novel method of paracrine signaling, potentially making microvesicles important components of stem cell niches. It also opens up the possibility of transferring siRNAs via microvesicles. The delivery of siRNAs by microvesicles may be particularly useful in the treatment of retinal disorders such as age related macular degeneration, since injection of naked siRNAs into the eye may have deleterious effects [19].

## Results

### ESMV RNA profile

**Figure 1:**
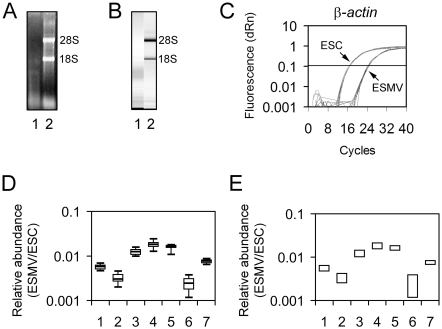
ESMVs contain RNA. ESMV total RNA lacks 28S and 18S rRNA and consists mostly of RNA below ∼2kb. (A) 1.2% denaturing agarose gel loaded with total RNA from ESMVs (lane 1) and ESCs (lane 2). (B) Digital gel images from capillary electrophoresis of total RNA from ESMVs (lane 1) and ESCs (lane 2). (C) Real time quantitative RT-PCR amplification curves for *β-actin* reveals much less expression in ESMVs compared with ESCs. (D) The relative abundance of several mRNAs in ESMVs compared with ESCs was determined by real time quantitative RT-PCR using the primers shown in Table 1. The mRNAs tested include *gata-4* (lane 1), *jag-1* (lane 2), *jag-2* (lane 3), *nanog* (lane 4), *oct-4* (lane 5), *wnt-3* (lane 6), and *β-actin* (lane 7). Box plot of relative abundance of all mRNA tested in ESMVs compared with ESCs (n = 8). The boxed area represents the mean±quartile and the whiskers extend out to the minimum and maximum values. Bootstrap ANOVA was performed and a significant difference was detected between all groups (*p*<0.0001). (E) The 95^th^ percentile confidence interval for each mRNA shown in (D) was determined and plotted on a bar graph. Non overlapping groups are significantly different from each other.

Although ESMVs have been reported to contain mRNA [6], little was known about their total RNA profile or the quality and types of RNA that they contain. Thus, we isolated mouse ESMVs and determined their total RNA profile by gel electrophoresis and capillary electrophoresis (Figures 1A and 1B). 4.77±0.7 μg (n = 10) of total RNA can be obtained over a 48 hour period from ESMVs released by 3.5×10^6^ ESCs plated on a T175 culture flask and cultured to ∼70% confluence in serum-free media. Unlike total RNA collected from ESCs, the total RNA from ESMVs does not contain 28S and 18S rRNA (Figures 1A and 1B). Instead, the majority of the total RNA in ESMVs is concentrated below 2 kb, which may represent mRNAs and small RNAs such as miRNAs.

### ESMVs contain mRNA

Consistent with previous studies [6], [10], [21], we detected the presence of mRNA in microvesicles. Purified polyA-containing RNA from ESMVs (0.59%±0.2% of total RNA, n = 4) was found to be less abundant than that present in ESCs (3.43%±0.6% of total RNA, n = 4). To compare the differences in transcript levels in ESMVs versus ESCs, we performed real time quantitative RT-PCR using primers shown in Table 1 and SYBR Green for detection. Interestingly, we observed an overall reduction of several orders of magnitude in the levels of all transcripts tested from ESMVs when compared with the levels of the same mRNAs in ESCs, including that encoding the cytoskeletal protein, *β-actin* (Figure 1C). Because of this disparity in expression levels, we could not use *β-actin* as a normalizer in comparative quantification experiments. Instead, we relied on adding equivalent amounts of RNA template in each experiment. The tested transcripts encoded transcription factors important for maintaining stem cell pluripotency (*oct-4*, *nanog*, and *gata-4*), cell surface ligands in the notch signaling pathway (*jag-1* and *jag-2*), and a secreted signaling protein (*wnt-3*) (Figure 1D). Bootstrap ANOVA suggested a significant difference between all groups, *F* = 3.33, *p*<0.0001. Further analysis using the 95^th^ percentile confidence interval calculated for each mRNA revealed differences between several groups (Figure 1E). Since we observed a reduction in abundance in all transcripts tested, we expected that mRNAs were overall less abundant in ESMVs compared with ESCs. However, the proportion of polyA RNA purified from ESMV and ESC total RNA (0.59% and 3.43%) reflected a 5.8-fold difference, whereas a much larger reduction of at least 55-fold was observed with all transcripts tested by quantitative RT-PCR.

**Table 1:** Primers used for RT-PCR.

Primer pairs	Sequence (5′–3′)	Amplicon (bp)
Oct-4 5′	GCCGGGCTGGGTGGATTCTC	271
	ATTGGGGCGGTCGGCACAGG	
Oct-4 3′	AGGCCCGGAAGAGAAAGCGAACTA	265
	TGGGGGCAGAGGAAAGGATACAGC	
GFP 5′	ATGGTGAGCAAGGGCGAGGAG	274
	CTTCGGGCATGGCGGACTTGA	
GFP 3′	TATCATGGCCGACAAGCAGAAGAACG	230
	CGGCGGCGGTCACGAACTCC	
GFP end-point	GTCCAGGAGCGCACCATCTTCTT	406
	CGGCGGCGGTCACGAACT	
Nanog	TCCAGAAGAGGGCGTCAGAT	283
	CTTTGGTCCCAGCATTCAGG	
β-actin	TTGTTACCAACTGGGACGACA	350
	TCTTCATGAGGTAGTCTGTCA	
Gata-4	AGGCGAGATGGGACGGGACACTAC	202
	CGCAGGCATTACATACAGGCTCAC	
Wnt-3	CTTCATGATCGCCGGCAAACTTC	189
	TGGGATGGAGCCGCAGAGCAGAG	
Jag-1	CAGTGCCTCTGTGAGACCAAC	193
	AGGGGTCAGAGAGACAAGCATG	
Jag-2	GCAAAGAATGCAAAGAAGCC	233
	TGGCTGCCACAGTAGTTCAGG	

### ESMVs contain protein

**Figure 2:**
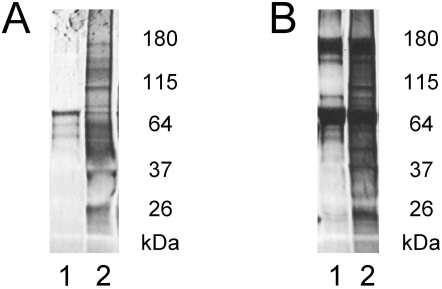
ESMVs contain protein. Insoluble and soluble protein fractions were isolated from ESMVs and separated on a Tris-glycine buffered 4–20% SDS-polyacrylamide gel, stained with SYPRO Ruby Red. (A) The insoluble protein fraction. Lanes: (1) control medium, (2) ESMVs. (B) The soluble protein fraction after precipitation with TCA. Lanes: (1) control medium, (2) ESMVs. Equivalent amounts of sample and control medium were processed.

Consistent with previous reports, abundant protein can be isolated from ESMVs. We obtained 25.8±4.5 μg (n = 3) of total protein from ESMVs released over a 48 hour period by 3.5×10^6^ ESCs originally plated on a T175 culture flask and cultured to ∼70% confluence in serum-free media. We digested microvesicles, pelleted the insoluble fraction, TCA precipitated the soluble proteins and separated both insoluble and soluble proteins by SDS-PAGE. Several distinct bands were present in ESMVs and not in the media controls suggesting that ESMVs may contain a subset of abundant proteins (Figure 2).

### ESMVs contain mRNA and protein expressed from a transgene in ESCs encoding GFP

**Figure 3:**
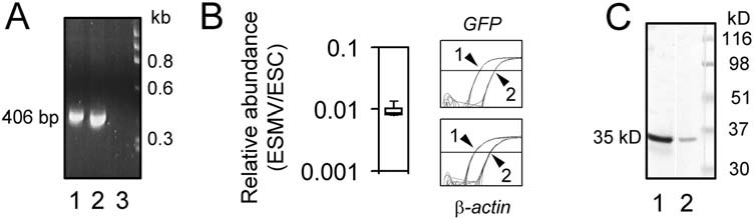
ESMVs contain GFP mRNA and protein expressed from a GFP transgene in ESCs. (A) 300 ng of ESMV RNA from an ESC line expressing GFP were used for RT, and 35 cycles of PCR amplification were performed with the GFP primers shown in Table 1. A 2% agarose gel was loaded with the RT-PCR products from ESMVs (lane 1), ESCs (lane 2), and a “no RT” control of ESMVs (lane 3). A 406bp band corresponding to the GFP amplicon is observed in both the ESMV and ESC lanes. (B) (Left) Box plot of relative abundance of GFP in ESMVs compared with ESCs (n = 8). (Right) Comparison of amplification curves for *GFP* (top) and *β-actin* (bottom) in ESCs (1) and ESMVs (2). Note that while quantitative RT-PCR was performed in the linear range of amplification, (panel B), the end-point PCR products shown in panel (A) are only qualitative and well outside of the linear range. (C) Immunoblot of an 8% urea-SDS polyacrylamide/Tris-glycine buffered gel loaded with 20 μg total protein/lane, using polyclonal anti-GFP antibody (1∶1000) and horse anti-rabbit secondary antibody (1∶5000). The secondary antibody was conjugated to alkaline phosphatase and visualized with BCIP/NBT. A single ∼35kD immunoreactive band corresponding to GFP in ESCs (lane 1) and ESMVs (lane 2) was detected.

We investigated whether ESMVs also contain mRNA and protein expressed from a GFP transgene present in ESCs. We isolated ESMVs from this GFP-expressing ESC line, performed RT-PCR with their RNA, and verified the presence of *GFP* mRNA (Figure 3A). The relative abundance of *GFP* mRNA in ESMVs versus ESCs was quantified by real time quantitative RT-PCR (Figure 3B) using primers shown in Table 1. The relative abundance of *GFP* mRNA in ESMVs was comparable to that of several of the endogenous transcripts tested (Figures 1D and 3B).

In order to determine if GFP (protein) was also present in ESMVs, proteins in ESCs and ESMVs were isolated, separated by PAGE, and transferred to a blot that was incubated with a polyclonal antibody against GFP. GFP was readily detected in ESMVs, although it was less abundant than in ESCs (Figure 3C).

### RNA integrity is maintained in ESMVs

**Figure 4:**
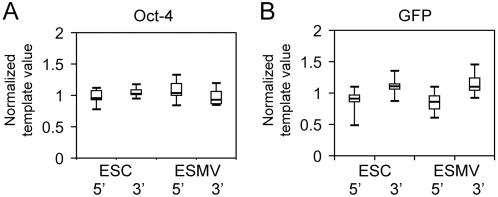
The RNA in ESMVs is not degraded. Real time quantitative RT-PCR was used to measure the level of degradation of *oct-4* and *GFP* mRNAs in ESMVs by comparing the 5′ and 3′ amplicon ratios of these transcripts in ESMVs with those in ESCs. Significant levels of degradation were not detected with either transcript. (A) Box plot of normalized 5′ and 3′ template values for *oct-4* mRNA in ESCs and ESMVs (n = 9). (B) Box plot of normalized 5′ and 3′ template values for *GFP* mRNA in ESCs and ESMVs (n = 12). The boxed area represents the mean±quartile and the whiskers extend out to the minimum and maximum values. Bootstrap t-tests were performed to compare the 5′:3′ ratios for each transcript in ESCs and ESMVs. No significant difference was detected between the ESC group and ESMV group for either transcript (*p*>0.1).

To confirm the integrity of the mRNA isolated from ESMVs, we used real time quantitative RT-PCR to determine the ratio of 5′ amplicons to 3′ amplicons of individual transcripts; ideally, the 5′∶3′ amplicon ratio should equal 1 if there is no degradation. However, RT inefficiencies and small errors in the calculated PCR primer efficiencies can generate 5′∶3′ ratios that deviate from 1. mRNA isolated from ESCs was used as control, and its quality was verified by determining the 28S∶18S rRNA ratio, which was >1.9 in all of our preparations (indicating minimal degradation). Real time quantitative RT-PCR was carried out to compare the 5′∶3′ ratio for ESMVs and ESCs for two transcripts, *oct-4* which has an average size (1.3 kb), and *GFP* which is exogenously expressed in these ESCs. Our results indicated that the mRNA in ESMVs is intact and not degraded (Figures 4A and 4B). Bootstrap t-tests used to compare the ESC 5′∶3′ groups with the ESMV 5′∶3′ groups for each mRNA showed no significant difference after 10,000 bootstrap resamplings for either *oct-4*, *p* = 0.495 or *GFP*, *p* = 0.517.

### ESMVs contain miRNAs

**Figure 5:**
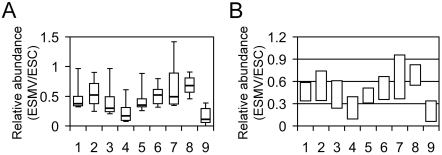
ESMVs contain miRNAs. The relative abundance of several miRNAs in ESMVs compared with ESCs was determined by real time quantitative RT-PCR. The miRNAs tested include *miR-16* (lane 1), *miR-21* (lane 2), *miR-22* (lane 3), *miR-290* (lane 4), *miR-291-3p* (lane 5), *miR-292-3p* (lane 6), *miR-294* (lane 7), *miR-295* (lane 8), and the small nuclear RNA, *RNU6b* (lane 9). (A) Box plots of relative abundance in ESMVs compared with ESCs (n = 9). The boxed area represents the mean±quartile and the whiskers extend out to the minimum and maximum values. Bootstrap ANOVA was performed and a significant difference was detected between all groups (*p* = 0.008). (B) The 95^th^ percentile confidence interval for each miRNA was determined and plotted on a bar graph. Non-overlapping groups are significantly different from each other. RNU6b is significantly less abundant than all miRNAs tested except *for miR-22, miR-290,* and *miR-291.* The majority of miRNAs tested do not differ significantly from one another except for *miR-295*, which is significantly more abundant than *miR-290* and *miR-291*.

Based on the total RNA profile (Figures 1A and 1B) and since mRNAs make up a small percentage of total RNA in ESMVs, a significant portion of RNA in ESMVs may be composed of small RNAs. We hypothesized that ESMVs contain abundant miRNAs in addition to mRNA. Using RT-PCR, we tested for the presence of a ubiquitous miRNA, *miR-16*, which is expressed in ESCs. Since mature miRNAs are very short transcripts, a strategy which uses a stem-loop RT primer was used [22]. Total RNA from ESMVs was isolated using two different methods: the mirVana miRNA isolation kit (Ambion) retains small RNAs, whereas the Nucleospin RNA II kit (Clontech) excludes small RNAs. Using equivalent amounts of starting total RNA template, we detected a *miR-16* signal only with the RNA samples containing small RNAs (not shown). We then performed real time quantitative RT-PCR, using Taqman Probes for detection, to quantify the abundance of several miRNAs in ESMVs (Figure 5A). We chose to profile the ubiquitously expressed *miR-16*, five ESC-specific miRNAs (*miR-290*, *miR-291-3p*, *miR-292-3p*, *miR-294*, and *miR-295*) [23], [24], and two miRNAs that are upregulated in ESCs undergoing differentiation (*miR-21* and *miR-22*) [23], [24]. We also included a small nuclear RNA (*RNU6b*), which is usually used as a normalizer. However, because of a large difference in *RNU6b* abundance in ESCs and ESMVs, we were unable to use it as a normalizer in these experiments. Instead, we performed our experiments using equivalent amounts of total RNA template. Our results showed a significant difference in relative abundance between several of the ESC-specific miRNAs. Bootstrap ANOVA showed a significant difference between all groups, *F* = 0.69, *p* = 0.008. Further analysis using the 95^th^ percentile confidence interval calculated for each miRNA revealed differences between some groups (Figure 5B). Non-overlapping groups at the 95% confidence interval are significantly different from one another. Thus, based on the 95^th^ percentile confidence interval, it appears that *RNU6b* is significantly less abundant than several of the miRNAs tested except *miR-22*, *miR-290*, and *miR-291*. The relative abundance of all tested miRNAs overlaps except for that of *miR-295*, which is significantly more abundant than *miR-290* and *miR-291* (Figure 5B).

### ESMVs fuse with other ES cells and transfer GFP

**Figure 6:**
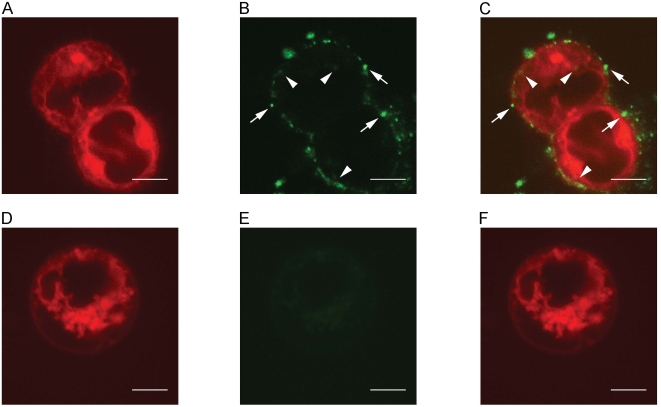
ESMVs transfer GFP. ESCs without the GFP transgene were labeled with DiD and then incubated with ESMVs containing GFP. All confocal images were taken using a 100×, 1.4 NA objective with the pinhole set to 1 airy unit. (A) DiD signal from ESCs incubated with ESMVs. (B) GFP signal from ESCs incubated with ESMVs. Arrows indicate punctate signal, likely representing docked vesicles. Arrowheads indicate diffuse signal, likely from the diffusion of GFP inside the cell or from the production of newly translated GFP. (C) Overlay of A+B. (D) DiD signal from control ESCs without ESMVs. (E) No GFP signal can be detected in the absence of ESMVs. (F) Overlay of D+E. All scale bars are 5 μm.

Previous studies demonstrated the transfer of endogenously expressed proteins and RNA from microvesicles to cells. [6], [10], [25]–[27]. To determine if ESMVs are capable of transferring exogenously expressed GFP, we incubated ESCs without the GFP transgene with ESMVs containing GFP. To better visualize the cellular borders, the ESCs were pre-incubated with the lipophilic dye, Vybrant DiD (Molecular Probes), prior to the addition of ESMVs. After a three-hour incubation with ESMVs, the cells were imaged by confocal microscopy. Cells that had been incubated with ESMVs were clearly seen using the appropriate DiD filter set (Figure 6A). The same cells were then imaged using the corresponding filter to visualize GFP labeling (Figure 6B). Multiple green vesicles can be seen docked on the ESCs (arrows) and several patches of diffuse GFP signal can also be seen inside the cells near the plasma membrane and in the cytoplasm (arrowheads, Figure 6B.). Figure 6C was obtained by merging the images of Figures 6A and 6B. Although the signal in the cytoplasm is not as robust as the signal from the docked vesicles, the docking and fusion of ESMVs to cells is highly efficient as the vast majority of cells contain docked vesicles. DiD-labeled control ESCs incubated without ESMVs (Figure 6D) did not show green fluorescence when visualized with the GFP filter set (Figure 6E). Figure 6F was obtained by merging the images of Figures 6D and 6E.

### ESMVs fuse with MEFs and transfer miRNAs

**Figure 7:**
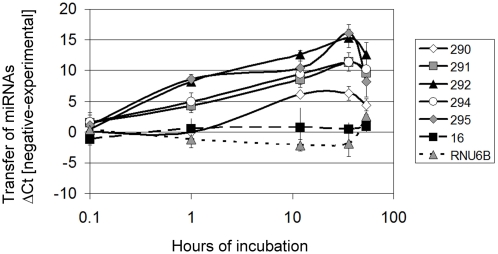
ESMVs transfer miRNAs. MEFs were incubated with ESMVs for 1, 12, 36, or 54 hours and transfer of miRNAs was determined by real time quantitative RT-PCR (n = 5). Time point 0 represents MEFs without ESMVs. The difference in Ct values between the negative control (MEFs alone) and each experimental group (miR-290, miR-291-3p, miR-292-3p, miR-294, miR-295, miR-16, and RNU6b) is shown. Positive values indicate transfer of miRNA.

The presence of miRNAs in ESMVs opens up the intriguing possibility that miRNAs may be transferred by microvesicles. We tested this hypothesis by incubating ESMVs with gamma-irradiated MEFs and compared the level of miRNAs found in MEFs alone with that of MEFs that had been incubated with ESMVs. The abundance of several miRNAs (miR-290, miR-291-3p, miR-292-3p, miR-294, and miR-295) increased in MEFs as early as 1 hour after incubation, suggesting transfer. However, this was not observed with all miRNAs tested. The ubiquitously expressed miR-16 (and the small nuclear RNA, RNU6b) did not transfer and remained near baseline at all time points tested (Figure 7). In general, peak transfer occurred at nearly the same time for all miRNAs capable of transfer, between 12–36 hours, but although at 54 hours the levels of the transferred miRNAs had not returned to baseline, they showed a significant downward trend. The miRNAs that appeared to transfer most efficiently were miRNAs found in abundance in ESCs but not in MEFs.

## Discussion

The ability of a cell to signal nearby cells, and to sense their local microenvironment, forms the basis for coordinated cellular activity in multicellular organisms and is particularly important during embryonic development. Early in development, embryo polarity is established so that the major axes (i.e. left-right, dorso-ventral, rostro-caudal) can be defined. The development and maintenance of polarity involves cell to cell communication [28] and embryonic stem cells in the early blastocyst undoubtedly communicate with one another. Traditional methods of intercellular communication mediated by cell-cell or cell-intercellular matrix contact involve gap junctions, secreted signaling molecules, and physical interaction of membrane proteins. An alternative method of intercellular communication involves microvesicles, which have been demonstrated to transport proteins and mRNA from cell to cell and also to transport surface ligands short distances to interact with cells [5], [6], [9], [10], [25]–[27]. We now report evidence of a novel method of intercellular communication mediated by the transfer of miRNAs by microvesicles. Our findings open up the possibility that miRNAs may also serve as signaling molecules allowing for coordinated intercellular regulation of gene expression. MiRNAs play important roles during embryonic development [29]–[33]. It is interesting to think that perhaps the intercellular transfer of specific miRNAs by microvesicles may help establish embryo polarity and tissue patterning by creating gradients of miRNAs.

Our results suggest that only certain miRNAs are efficiently transferred to MEFs. Perhaps the transfer of miRNAs is an active process, possibly regulated by proteins found in microvesicles or receptors found on recipient cells. Alternatively, the abundance of miRNAs may be tightly regulated by specific nucleases such that miR-16 levels inside MEFs are kept within a specific range, but the ESC specific miRNAs are not. In our experiments, we tested the ability of ESMVs to transfer miRNAs to growth-arrested MEFs. We can speculate that the efficiency of transfer may be different, perhaps even enhanced, with rapidly dividing or proliferating cells such as those found in a developing blastocyst. However, we have not tested this hypothesis at this time. The ability of microvesicles to transfer miRNAs is a novel finding which expands upon the roles that miRNAs are thought to play.

Stem cell and tissue specific microvesicles are interesting candidates for novel signaling factors in stem cell niches [34]. They are capable of carrying mRNAs, miRNAs, and proteins short distances and would be ideal paracrine factors in the microenvironments of stem cells. Ratajczak *et al*. [6] have already demonstrated that ESMVs are capable of enhancing the proliferation of hematopoietic progenitors. Our study shows that microvesicles released from ESCs are capable of transferring not only mRNA and protein, but also miRNAs to cells. Further study of microvesicles and their contents, released from other stem and progenitor cells may help elucidate the exact role that microvesicles play within stem cell niches. If microvesicles are indeed mediators of signaling in stem cell niches, they may be useful targets to reactivate or modulate the behavior of quiescent stem cells following injury.

The ability of microvesicles to transfer RNA and protein, and to act as paracrine factors raises very exciting possibilities for therapeutic uses. Cells engineered to express mRNA, siRNA, or protein may be capable of delivering these macromolecules to local cellular environments via microvesicles. These engineered cells can be encapsulated to provide sustained local delivery. Since current techniques for gene transfer use viral or synthetic agents as delivery agents, their replacement by microvesicles released from autologous transplants of engineered cells will offer the advantage of a virus-free approach and make the prospects of gene therapy safer.

## Materials and Methods

### Ethics Statement

All experiments involving mice were carried out according to protocols approved by the UCLA Animal Research Committee and in accordance with the ARVO Statement for use of animals in Ophthalmic and Vision Research.

### Isolation of ESMV RNA and RT-PCR

ESCs expressing the GFP transgene under the control of the chicken β-actin promoter were obtained from the laboratory of Timothy Ley (Washington University, Saint Louis). We generated a second ESC line derived from C57Bl/6 mice. ESCs were maintained in feeder-free conditions using ESGRO complete (Chemicon) serum-free medium supplemented with 1–5% FBS to enhance proliferation.

To prepare for ESMV collection, ESCs were expanded under serum-free and feeder-free conditions. 3.5×10^6^ cells were plated on gelatin-coated T175 culture flasks. 48 hours after plating, the media was collected and spun at 3500g for 30 minutes at 4°C to pellet debris and fragmented cells. The supernatant was carefully transferred to an ultracentrifuge tube and spun at 100,000g for 2 hours in a SW 28 or SW 40ti rotor at 4°C to pellet the microvesicles. RNA was immediately frozen at −80°C in lysis buffer from the mirVana miRNA isolation kit (Ambion), which contains guanidinium thiocyanate, so that multiple samples could be pooled together. When the desired number of samples was collected, the RNA was isolated following Ambion's protocol, which retains small RNAs. To prepare samples excluding small RNAs, we used the Nucleospin RNA II kit (Clontech) and followed the manufacturer's protocol. The RNA was quantified in a Nanodrop ND-1000, analyzed by gel electrophoresis or bioanalyzer (Agilent), and treated with TURBO DNAse (Ambion) prior to further manipulation. RT-PCR was performed with Improm-II RT (Promega) and the cDNA was amplified with recombinant Taq polymerase (New England Biolabs). 35 cycles of PCR in a non-linear, saturating range of amplification were carried out with an annealing temperature of 60°C and an extension time of 30 seconds using standard cycling conditions. PCR products were analyzed by agarose gel electrophoresis. The Dynabeads mRNA Purification Kit (Dynal Biotech) was used to enrich the polyA-mRNA from total RNA.

### Real time quantitative RT-PCR

Real time quantitative RT-PCR was performed using an MX3000p instrument (Stratagene). DNase-treated total RNA was reverse transcribed using Improm-II RT for mRNA or MuLV RT (Applied Biosystems) for miRNAs. Detection with SYBR Green (Stratagene) was used for mRNA assays and Taqman probes (Applied Biosystems) for miRNA assays.

For all SYBR Green assays, standard curves were generated for each primer set to determine their efficiency, and dissociation curves were generated to detect non-specific amplification products and primer-dimers. PCR products were also analyzed by agarose gel electrophoresis to confirm that a single PCR product was produced. A set of “no RT” controls were performed with each batch of RNA, and no template controls were included with each experiment. Standard curves were used to establish an initial template quantity (absolute quantification) in determination of 5′:3′ ratios. The data was normalized against the composite median of both 5′ and 3′ initial template values in order to plot the experiments on the same scale, without altering the ratios. A normalizer was not necessary since the same transcript from the same sample was amplified by two sets of primers (5′ or 3′). Standard cycling conditions were used with an annealing temperature of 60°C.

Comparative quantification of different mRNAs was performed without a normalizer and thus, the 2^−ΔΔCt^ method of quantification [35] was not used. In order for a normalizer to be valid, it must have the same copy number in both the control (calibrator) sample and test sample (unknown), and we did not have any mRNA with this characteristic. Thus, to compare the relative abundance of mRNA transcripts in ESCs with ESMVs, we relied on using equivalent amounts of ESC and ESMV total RNA in each experiment. The ESC samples were set as calibrators for the ESMV samples and corrections for primer efficiency were performed using the MX Pro software (Stratagene). Comparative quantification of miRNAs with Taqman probes was performed using Taqman miRNA assays (Applied Biosystems) according to the manufacturer's protocol. The assays used were: hsa-miR-16, hsa-miR-21, hsa-miR-22, mmu-miR-290, mmu-miR-291-3p, mmu-miR-292-3p, mmu-miR-294, mmu-miR-295, and the small nuclear RNA, RNU6b. We were unable to find a suitable normalizer, thus the same method of quantification that was used for mRNAs was also used for miRNAs.

Table 1 lists the non-commercial PCR primers used. For quantitative comparison, the *oct-4* and *GFP* 5′ primer sets were used. The commercially available primer sequences for the Taqman assays can be downloaded from the manufacturer's website (http://www3.appliedbiosystems.com/cms/groups/portal/documents/generaldocuments/cms052133.xls).

### Isolation of ESMV protein and immunoblot

To isolate crude protein, the final ESMV pellet was incubated on an agitator with digestion buffer (2% Triton X-100, 0.3% CHAPS, 1mM PMSF in PBS) at 4°C to disrupt the membranes. The insoluble fraction was pelleted by centrifugation at 20,000×g for 1 hour at 4°C. The soluble fraction was collected and precipitated with 20% trichloroacetic acid (TCA), washed twice with ice-cold acetone and separated on a Tris-glycine buffered 4–20% SDS-polyacrylamide gel.

To detect GFP protein, the ultracentrifuged ESMV pellet was resuspended in ice-cold cell disruption buffer from the mirVana Paris kit (Ambion) and the total protein was quantified using the Bradford assay. Crude protein preparations were stored frozen at −80°C or used immediately for analysis. Prior to loading, the samples were heated to 87–90°C for 5 minutes in sample buffer containing 0.2M DTT and 1.8M beta-mercaptoethanol. Total protein (20 μg per sample) was separated on a 8%T/3%C, 6M urea, SDS-polyacrylamide gel using a Tris/glycine buffer system, and transferred to a PVDF membrane overnight. GFP was detected with 1∶1000 diluted rabbit anti-GFP polyclonal antibodies (Molecular Probes) and 1∶5000 diluted horse anti-rabbit IgG conjugated to alkaline phosphatase (Vector Labs). Immunoreactive bands were visualized with 5-bromo-4-chloro-3′-indolylphosphate p-toluidine salt and nitro-blue tetrazolium chloride (BCIP/NBT).

### Transfer of GFP to ES cells

Immediately after passaging, 200 μl of ESCs (∼10^6^ cells/mL) were transferred to a culture tube in serum-free medium with 1 μl of Vybrant DiD (Molecular Probes). The cells were incubated for 20 minutes at 37°C, and then washed 2× with pre-warmed DMEM. After the last wash, the cells were resuspended in 200 μl serum-free medium and separated into two tubes. An additional 100 μl of serum-free medium was added to one tube serving as control and 100 μl of ESMVs containing GFP (collected from 3-4 T175 flasks) were added to the second tube. Both tubes were incubated at 37°C for 3 hrs prior to imaging the cells on a Leica TCS-SP inverted confocal microscope. Images were taken with a 100×, 1.4 NA plan apochromatic objective. Excitation was achieved with the 488nm laser line for GFP and the 633nm laser line for DiD. The spectral detector was set to 498–531nm for GFP and 650–714nm for DiD detection. All images were taken with the pinhole set to 1 airy unit. The DiD image was overlayed on the GFP image after digitization using the Leica confocal software or Adobe Photoshop CS2.

### Transfer of miRNAs to embryonic fibroblasts

Approximately 1.6×10^5^ cells/well of gamma-irradiated MEFs were pre-plated on 24 well plates at least 2 days prior to initiation of the experiment. ESMVs isolated from 6 T175 flasks (as described above) were divided evenly between all samples and co-incubated with the pre-plated MEFs. Growth-arrested MEFs were used to maintain a constant ratio of ESMVs to MEFs between each experiment since we do not know if ESMVs have any effect on the growth or proliferation of MEFs. Each experimental series included a negative control (MEF only) and 4 experimental time points (MEF+ESMVs). We collected MEFs after 1, 12, 36, and 54 hours. To collect the cells while removing free-floating ESMVs, MEFs were washed with PBS twice prior to dissociation. MEFs were then enzymatically dissociated from the plates and washed two more times with PBS. After each wash, cells were spun down at 3500×g for 5 minutes so that any residual ESMVs would float in the supernatant and be discarded. We isolated the MEF total RNA and performed real time quantitative RT-PCR with a subset of miRNAs using the protocol described above. As an indirect measure of miRNA transfer, we determined the difference in Ct values between the negative control and each experimental time point; a positive value indicated transfer. If no signal was detected, a Ct value of 40 was assigned to the sample.

### Bootstrap statistics

Bootstrap statistics emulates real life by generating random and independent data points from the original data set using computer-intensive programs. All statistical analyses were performed using Excel (Microsoft) and the Excel Add-in from Resampling Stats software (Statistics.com) using standard methods [36]. For bootstrap t-tests comparing the 5′∶3′ ratio of ESCs with ESMVs, the initial template values were normalized as described above. Pseudogroups of the normalized values were randomly generated with replacement and the 5′∶3′ ratios were calculated from the pseudogroup means. The difference between the 5′∶3′ ratios for ESMVs and ESCs was resampled 10,000 times and binned to determine the 95^th^ percentile confidence intervals for acceptance of the null hypothesis. For bootstrap ANOVA, the pseudogroups were generated from the relative quantity of each transcript or miRNA tested. The medians of these pseudogroups were resampled 10,000 times and binned to determine the 95^th^ percentile confidence intervals and for calculating the F-ratio.
